# A Limited Survey of Aflatoxins in Poultry Feed and Feed Ingredients in Guyana

**DOI:** 10.3390/vetsci4040060

**Published:** 2017-11-24

**Authors:** Donna M. Morrison, David R. Ledoux, Lambert F. B. Chester, Coretta A. N. Samuels

**Affiliations:** 1 Faculty of Agriculture and Forestry, University of Guyana, Turkeyen, P.O. Box 10-1110, Guyana; Lambert.chester@uog.edu.gy (L.F.B.C.); coretta.samuels@uog.edu.gy (C.A.N.S.); 2 Department of Animal Sciences, University of Missouri-Columbia, Columbia, MO 65211, USA; ledouxD@missouri.edu

**Keywords:** aflatoxins, enzyme-linked immunosorbent assay, high-performance liquid chromatography, finished poultry feed, poultry feed ingredients

## Abstract

A study was conducted to determine the presence of aflatoxins in finished poultry feed from manufacturing companies, feed ingredients, and poultry feed at the point of sale. Two collections were made. In the first collection, samples of the finished feed and feed ingredients were analyzed by high-performance liquid chromatography (HPLC). For the second collection, all samples were analyzed by ELISA while a subset was analyzed by HPLC. Of the 27 samples of finished feed, five samples had aflatoxin concentrations greater than the United States Food and Drug Administration (USFDA) and European Union Commission (EUC) maximum tolerable limit of 20 µg/kg, while for the feed ingredients, three of the 30 samples of feed ingredients exceeded the limit. Of the 93 samples of finished feed purchased from retailers, five samples had aflatoxin concentrations greater than the maximum tolerable limit. This survey indicates that most of the samples were below the maximum regulatory limit and maintained quality up to the point of sale for 2015 and 2016. However, given that some samples were above the limit, there is a need to monitor the production and marketing chain to ensure that the quality of the finished feed is not compromised.

## 1. Introduction

Guyana is located in the north of South America, and is divided administratively into 10 regions numbering from 1 to 10. Poultry production in Guyana has increased significantly over the last 10 years. There was an increase in the production of poultry meat, mainly chicken, from 25.2 million kilograms (kg) in the year 2007 to 32.8 million kilograms (kg) in 2016, while egg production increased from 9.8 million to 22.4 million for the same period [1].

In Guyana, there are two basic types of poultry production systems, homestead subsistence farms and larger commercial operations. Farmers either purchase feed directly from the manufacturers or from retailers. The retail outlets sell mainly chick starter and chick grower feed. The chick starter is fed to chicks for the first six weeks after hatching, followed by chick grower for the next four weeks. For all finished feed, crude protein was the ingredient with the highest percentage. Crude protein sources included corn, rice bran, soybean meal, protein meals, and copra meals. This was supplemented with vitamins and minerals. These finished products and feed ingredients are stored under various environmental conditions and may become contaminated with microorganisms including fungi and their metabolites. The most important genera of fungi in food and feed are *Aspergillus*, *Fusarium*, and *Penicillium*, and under the right circumstances they can produce mycotoxins—aflatoxins, ochratoxins, zearalenone, trichothecenes, and fumonisins [2].

The presence of fungi and their metabolites can result in severe economic losses to farmers and retailers, and are a potential threat to consumers. While there is no literature on aflatoxin contamination of poultry feed in Guyana, *Aspergillus* and aflatoxin B1 have been found in samples of poultry feeds and feed ingredients in the neighboring countries of Trinidad and Brazil [3,4]. Aflatoxins are secondary metabolites of the fungi *Aspergillus flavus* and *Aspergillus paraciticus*. There are four major aflatoxins, B1, B2, G1, and G2, with B1 usually being the aflatoxin of the highest concentration in contaminated feed and food [5]. Aflatoxins are rated as the most toxigenic of all mycotoxins in animals [6]. At the embryonic stage of development, the oral LD50 (median lethal dose that kills 50% of a tested population after a specified test duration) is 0.3 to 5 mg/kg of body weight, whereas for young chicks it is 6.5 to18 mg/kg body weight.

There are multiple effects on poultry fed aflatoxin-contaminated feed, and almost all systems in the body are affected. For example, deformities caused by interference in bone metabolism resulted in decreased bone strength and a reduction in bone diameter [7,8]. Aflatoxin caused changes in the arrangement of the muscles, resulting in decreases in dressed weight and breast yield, while back, wing, drum, and thigh weight increased [9]. This can result in a significant impact on the marketable quality of the poultry meat.

Apart from the health and quality of the chickens, there are concerns that aflatoxin contaminants may pass from processed chicken products to humans consuming such products. Several studies have noted the presence of aflatoxin residues in the eggs and muscles of chickens [10,11,12] consuming aflatoxin-contaminated feed. Therefore, at the production level, management should have systems in place to ensure that the feed ingredients are safe and that careful sanitation is maintained at all levels to prevent contamination during production. It is also important that retailers sell and store their feed in a clean and healthy environment.

The objectives of this study were to determine: (1) the aflatoxin concentration in feed ingredients as well as finished poultry feed manufactured and sold in Guyana; and (2) whether the concentrations of aflatoxins were potentially harmful to the health of poultry and humans.

## 2. Materials and Methods

There were two collection periods in this study, one in 2015 and the other in 2016. Samples from the first collection period were analyzed by HPLC while those from the second collection were analyzed by ELISA, and a subset was analyzed by HPLC for confirmation.

### 2.1. Sample Collection and Preparation

In the first collection period in 2015, 10 composite samples of poultry feed were collected from the three major manufacturing companies in Guyana. In the presence of the principal researcher, all three companies provided freshly made samples. Two companies provided one composite sample each of chick starter and chick grower. The third company provided two samples of chick starter and chick grower and these were analyzed separately. The third company also supplied composite samples of broiler finisher and layer feed. In the follow-up collection in 2016, 17 composite samples were collected from the same three manufacturing companies. At least one composite sample of chick grower and one composite sample of chick finisher were collected from each company. In addition, one of those companies provided pullet developer and finisher feed while another supplied egg ration, growing ration, and finisher ration. In the first collection, feed ingredients of corn and copra were obtained from two companies, while in the second collection, bran was obtained from one manufacturer and additional bran and wheat middling samples were purchased from retailers. The collection of finished chicken feed from retailers focused on the five more populated areas that contained about 80% of Guyana’s population, where it is expected that there is a high volume of poultry consumption. Ten samples each of chick starter and chick grower feed were collected from Regions 4, 5, and 6, six samples of chick starter and eight samples of chick grower were collected from Region 2, and 10 samples of chick starter and nine samples of chick grower were collected from Region 3, totaling 93 samples (46 chick grower and 47 chick starter). All samples were finely ground with a Stein M-2 sample mill for 1 min to an average particle size of less than 0.4 mm.

### 2.2. High-Performance Liquid Chromatographic Determination of Mycotoxins

All reagents were of analytical grade and solvents were HPLC standard. For extraction, 100 mL of acetonitrile:water (7:3) were added to 25 g of the finely ground samples in a propylene screw cap bottle and shaken for 30 min on a Thermo-Fisher Scientific Rotator Shaker (Waltham, MA, USA). PuriToxSR aflatoxin cleanup columns (TC-M160), purchased from Trilogy Analytical laboratory (Washington, MO, USA), were placed into Corning^®^ polypropylene 15 mL Falcon centrifuge tubes (Sigma-aldrich, St. Louis, MO, USA). After shaking, the solids were allowed to settle and 4 mL of the supernatant was passed through the column and collected in Falcon tubes. The tubes were vortexed and 200 µL was added to 800 µL water in an autosample vial. The sample extracts were analyzed for aflatoxins as AFB1, AFB2, AFG1, and AFG2 by HPLC. These were individually determined after post column derivatization with bromine using a Kobra cell TM (R-Biopharm, Rhone Ltd., Glasgow, UK).

The HPLC system (Hitachi Elite and La Chrom, Technologies America, Schaumburg, IL, USA) consisted of a Model L-2130 pump, a model L-2485 fluorescence detector, and a model L-2200 autosampler with Hitachi D-2000 Elite software on a microcomputer. The column used was a Phenomenex 100 × 4.6 reversed phase hyperclone 3 µm C18BDS at a flow rate of 0.9 mL/min. The mobile phase was methanol:acetonitrile:water (1:1:4) with the addition of 1.2 g potassium bromide and 360 µL of nitric acid. The chromatographic parameters were: flow rate of 0.9 mL/min, injection volume of 50 µL, with the column at room temperature. Samples were analyzed with Kobra cell (R-Biopharm Rhone Ltd., Glasgow, UK) post column derivatization with fluorescence detection (λex. = 360 nm, λem. = 440 nm). Aflatoxins were quantified using retention time, peak area, and external calibration curves. The approximate retention times for AFB1, AFB2, AFG1, and AFG2 were 14.2 min, 10.4 min, 9.5 min, and 7.5 min, respectively. Linearity of the HPLC method was checked from correlation coefficients of the calibration curves using AFB1 (40–800 µg/kg), AFB2 (10–200 µg/kg), AFG1 (40–800 µg/kg), and AFG2 (10–200 µg/kg). The accuracy of the method was evaluated by recovery of a spike of known concentration of standards. The limit of detection and the limit of quantitation were 1 µg/kg and 5 µg/kg, respectively. This method was adapted from that previously applied in the analysis of rice [13].

### 2.3. Analysis by Enzyme-Linked Immunosorbent Assay (ELISA)

For the second collection, all samples were analyzed according to a MaxSignal^®^ Total Aflatoxin ELISA test kit (Bioo Scientific, Austin, TX, USA). Five grams of the samples were placed in a centrifuge tube and aflatoxins were extracted by adding 25 mL of 70% methanol, shaking on a rotary shaker for 20 min followed by centrifuging at 4000 rpm for 10 min. One (1) mL of the supernatant was diluted with 1 mL of Phosphate Buffer Saline (PBS) and mixed by vortexing. Fifty (50) µL of the diluted supernatant was placed in duplicates in two microwells. One hundred (100) µL of aflatoxin B1-HRP (Horseradish phosphatase) conjugate base was added to each microwell and mixed by rocking the plate for 1 min. The plate was then incubated for 30 min at room temperature, after which the solution was discarded. The plate was then washed by adding 250 µL wash solution, followed by shaking for 10 s. The washing solution was then discarded. This washing process was repeated two more times. After the third washing, the plate was dried, 100 µL of TMB substrate was added to each microwell, mixed, and then incubated for 15 min at room temperature. After incubation, 100 µL of the stop buffer was added to the solution to stop the enzyme reaction. A calibration curve was prepared with aflatoxin B1 standards of 0.05 ng/mL, 0.1 ng/mL, 0.2 ng/mL, 0.4 ng/mL, and 0.8 ng/mL. Each concentration was duplicated in two microwells. The plate was then read at 450 nm wavelength. The detection limit for feeds was 0.5 µg/kg. The intraassay coefficient of variation was calculated using the five standards. A subset of the samples was analyzed by HPLC using the method outlined above.

### 2.4. Statistical Analysis

Descriptive statistics were determined and the data collected from HPLC and ELISA were analyzed as a one-sample *t*-test with 20 µg/kg as the maximum tolerable limit as established for feeds by References [14,15]. All statistical analyses were conducted using the Statistix^®^ 10 software package (Statistix Analytical Software, Tallahassee, FL, USA).

## 3. Results and Discussion

A total of 27 feed samples were collected from the three major feed manufacturing companies in Guyana. Total aflatoxin concentration in these samples ranged from “non-detected (nd)” to 352.1 µg/kg. Of the five samples with aflatoxin greater than the maximum regulatory limit for feed of 20 µg/kg, four of the samples were within the range of 20 µg/kg to 40 µg/kg. The descriptive statistics show a relatively high standard deviation as a result of the outlier value of 352.1 µg/kg, but most of the values were between ‘non-detected (nd)’ and 3.43 µg/kg (Table 1).

The one-sample *t*-test comparing whether the samples were greater than the standard of 20 µg/kg resulted in a *t*-value of −0.13, *df* = 26 and *p* = 0.55 indicating that the levels of aflatoxins from the samples were not statistically higher than the regulatory limit. However, from a public health perspective, the five samples had unsafe levels of aflatoxins. These results are similar to those reported in Brazil, where 97% of the samples analyzed were below the EUC maximum limit [16], but differed from the results obtained in Argentina, where 48% of the analyzed samples had aflatoxins ranging from 17 to 197 µg/kg [17].

The concentrations are not likely to affect the health of broilers raised to 8 weeks of age, given the LD50 range of 5.3 ± 1.0 to 9.7 ± 0.8 mg/kg body weight. Further, the threshold values which affect growth rate and the weight of the liver, pancreas, and spleen as observed by Reference [18] are much higher that the concentrations determined in the finisher feed. The sample that was at the upper limit for finisher feed (352.1 µg/kg) could potentially affect the health of poultry, but this was an isolated case that needs further investigation.

The presence of aflatoxin in feed ingredients can influence the quality of the finished feed, and this can subsequently affect the health of animals and humans. Therefore, it is critical to determine the presence of pathogens and potential sources of contamination during the manufacturing process. In Guyana, the feed manufacturing companies import feed concentrate, and other ingredients such as corn, copra, bran and wheat middling are added to prepare the finished feed. Corn is imported, bran and copra are produced in Guyana, while wheat middling is a product of the flour produced in Guyana from imported wheat. Both finished poultry feed and feed ingredients were found to be contaminated with aflatoxins in Pakistan [19]. In Venezuela, 17 of the 40 samples of poultry feed ingredients tested were contaminated with aflatoxins, with the highest concentration of aflatoxin found in corn flour [20]. In this study, of the 30 samples of feed ingredients, three samples (corn, copra, and bran) had aflatoxin concentrations above the permissible level, but most of the samples were within the range of “non-detected (nd)” to 6.96 µg/kg (Table 1). The one-sample *t*-test indicates that most of the values were below the regulatory limit, with the mean of all values less than 20 µg/kg, *t*-value = 10.09, *df* = 29, and *p* = 1.00 indicating that the levels of aflatoxins from the samples were not statistically higher than the regulatory limit. Corn, one of the main ingredients in poultry feed, when made moldy and fed to broiler chicks, caused the chicks to develop severe leg deformities, and their caeca was distended with brown material and there were lesions on the sciatic nerve [21]. Higher levels of aflatoxins in corn were observed in humid environments [22,23]. Copra, a product from coconuts, is also among the protein sources used in poultry feed, and is recognized by the USFDA as a source of aflatoxin [24].

The transport of finished feed from the mills to retailers and the method of storage are potential areas for fungal growth. In Guyana, feed companies are located near the capital city and feed is transported by trucks to other parts of the country. Pressure on the feed during transport reduces the feed into smaller particle sizes, which encourages the growth of fungus [25]. In addition, exposure to high relative humidity and long periods of storage are directly related to fungal contamination. It would have been interesting to collect different types of feed samples for the analysis; however, most retailers sell only chick starter and chick grower because these feeds are most profitable. Most poultry producers are involved in rearing birds to produce the meat for sale to their surrounding community.

The samples collected from retailers consisted of 46 chick grower and 47 chick starter feeds. Among other differences between the samples, the starter feed contained higher concentrations of crude protein. However, statistically there was no difference between the two types of samples. Of the 93 samples, five had aflatoxin levels above the regulatory limit, ranging from 21.14 µg/kg to 30.82 µg/kg (Table 1). The one-sample *t*-test indicates that most of the values were below the regulatory limit with mean values less than 20 µg/kg, *t*-value = −16.31, *df* = 92, and *p* = 1.00 indicating that the levels of aflatoxins in the samples were not statistically higher than the regulatory limit.

A subset of 12 samples analyzed by ELISA was analyzed by HPLC for confirmation. The mean of the ELISA values was 4.63 µg/kg and that for HPLC was 1.8 µg/kg (Table 2). The Pearson correlation between the values was 0.62. Analysis of animal feed samples for AFB1 with HPLC and ELISA determined that both methods are reliable, but HPLC has a higher sensitivity and specificity than ELISA [26]. HPLC has high-performance separation. However, the disadvantage of ELISA is the possibility of cross-reaction, which may account for the higher number of samples detected with aflatoxins [27].

## 4. Conclusions

The survey indicates that most of the samples of feed and feed ingredients had aflatoxin concentrations below the maximum regulatory limit. However, there appears to be some cases of high levels of aflatoxin. Therefore, feed manufacturers should consider investing in a rapid test for aflatoxin so as to ensure that the ingredients and finished feed are of the expected quality. If possible, they should monitor the feed as it goes through the marketing/supply chain.

## Figures and Tables

**Table 1:** Descriptive statistics for poultry feed collected from companies; feed ingredients and chick starter and grower collected from retail outlets.

Type of Samples	Number of Samples	Total Aflatoxin Concentration (µg/kg)			Number of Samples above the Regulatory Limit of 20 µg/kg
		Mean ± SD	Minimum	Maximum	
Poultry feed from manufacturers ^a,b^	27	27.38 ± 82.12	nd	352.10	5
Poultry feed Ingredients ^a–c^	30	3.81±6.20	nd	30.49	3
Chick Grower from retailers ^b^	46	9.26±7.14	nd	23.6	2
Chick Starter from retailers ^b^	47	10.41±6.19	nd	30.82	3

Nd—non-detected (detection limit—0.5 µg/kg). These were not included in the statistical analysis.

^a^ Analyzed by HPLC; ^b^ Analyzed by ELISA; ^c^ Corn, copra, wheat middling, and bran.

**Table 2:** Comparison between the results for total aflatoxins in poultry feed samples analyzed by ELISA and HPLC.

Source	ELISA-Total Aflatoxins ^1^ (µg/kg)	HPLC-Total Aflatoxins ^1^ (µg/kg)
Bran	2.3	0.45
Bran	12.02	5.8
Chick Starter	3.29	nd
Chick Starter	2.87	0.27
Chick Starter	7.81	6.56
Chick Starter	4.52	nd
Chick Starter	3.98	0.69
Chick Starter	3.67	0.17
Chick grower	6.28	nd
Chick grower	nd	0.11
Chick grower	4.25	0.3
Chick grower	4.6	7.42
**Mean**	**4.63**	**1.81**

^1^ Total aflatoxins = AFB1, AFB2, AFG1, and AFG2. nd—non-detected.
